# Early Hippocampal Synaptic Loss Precedes Neuronal Loss and Associates with Early Behavioural Deficits in Three Distinct Strains of Prion Disease

**DOI:** 10.1371/journal.pone.0068062

**Published:** 2013-06-26

**Authors:** Kathryn J. Hilton, Colm Cunningham, Richard A. Reynolds, V. Hugh Perry

**Affiliations:** 1 School of Biological Sciences, Southampton General Hospital, Southampton, United Kingdom; 2 Trinity College Institute of Neuroscience & School of Biochemistry and Immunology, Trinity College Dublin, Dublin, Ireland; University of Victoria, Canada

## Abstract

Prion diseases are fatal neurodegenerative diseases of the CNS that are associated with the accumulation of misfolded cellular prion protein. There are several different strains of prion disease defined by different patterns of tissue vacuolation in the brain and disease time course, but features of neurodegeneration in these strains have not been extensively studied. Our previous studies using the prion strains ME7, 79A and 22L showed that infected mice developed behavioural deficits in the same sequence and temporal pattern despite divergent end-stage neuropathology. Here the objective was to address the hypothesis that synaptic loss would occur early in the disease in all three strains, would precede neuronal death and would be associated with the early behavioural deficits. C57BL/6 mice inoculated with ME7, 79A, or 22L-infected brain homogenates were behaviourally assessed on species typical behaviours previously shown to change during progression and euthanised when all three strains showed statistically significant impairment on these tasks. A decrease in labelling with the presynaptic marker synaptophysin was observed in the stratum radiatum of the hippocampus in all three strains, when compared to control animals. Negligible cell death was seen by TUNEL at this time point. Astrocyte and microglial activation and protease resistant prion protein (PrP^Sc^) deposition were assessed in multiple brain regions and showed some strain specificity but also strongly overlapping patterns. This study shows that despite distinct pathology, multiple strains lead to early synaptic degeneration in the hippocampus, associated with similar behavioural deficits and supports the idea that the initiation of synaptic loss is a primary target of the misfolded prion agent.

## Introduction

Prion diseases are fatal neurodegenerative diseases of mammals, including humans. The relationship between degeneration of particular neuronal circuits and the appearance of disease signs in mice, and indeed symptoms in humans, is largely unexplored. There is evidence to suggest that neuronal loss is a relatively late event and that this is preceded by synaptic loss [1,2]. Furthermore, there is evidence that reversing prion-associated toxicity in the early stages of disease, by conditional knockout of the normal cellular form of the prion protein, PrP^c^, can return mice to an ‘asymptomatic’ state [3]. However, it remains unclear as to which neurons or neuronal pathways are first targeted and to what extent this is prion strain-dependent.

Different strains of murine prion disease have been characterised on the basis of brain tissue vacuolation pattern and incubation time [4,5]. The end stage pathologies of these strains are diverse and involve many different regions of the brain. The strains used in this study have distinct vacuolation profiles [4] indicating distinct neuropathology. The primary features of these strains are marked neuronal loss in the CA1 field of the hippocampus in ME7, prominent vacuolation of the white matter in 79A and cerebellar Purkinje cell death in the 22L strain. Despite these divergent end stage pathologies, mice infected with each strain show the same sequence of onset of behavioural deficits in a battery of tasks [6]. The sequence commences with a decline in spontaneous species-typical behaviours such as burrowing, glucose consumption and nesting, progressing to cognitive deficits and hyperactivity in open field assessment and later encompassing deficits in co-ordination, balance and muscle strength [1,6–9]. The similarity in the sequence of behavioural change in ME7, 79A and 22L indicates that there may be early common pathways that become dysfunctional or degenerate and that certain populations of neurons could be particularly vulnerable, but this remains little investigated.

Hippocampal neuronal death has been demonstrated in various prion strains but this is clearly a late stage phenomenon [1,2,6] and is not found at 13 weeks post ME7 inoculation when behavioural deficits first appear [1]. However, disturbances in synaptic proteins, loss of synapses and dendritic alterations including the loss of spines, particularly in the stratum radiatum of CA1, have been reported at 40-55% disease duration [1,2]. Brain biopsies and post mortem tissue from patients with prion disease have synaptic disorganisation and loss, showing an accumulation of subcellular organelles, dark synapses and a decrease in synaptophysin immunoreactivity [10–12]. Furthermore there is clear electron microscopic evidence that presynaptic terminals begin to degenerate and are subsequently surrounded or engulfed by post-synaptic densities [13]. Given the convergence of data on presynaptic terminal loss as an early event in the degenerative process we investigated whether there is early synaptic loss that is common to ME7, 79A and 22L strains, which are known to show divergent end stage pathology. To investigate this, mice were behaviourally monitored longitudinally until they showed statistically significant deviations from control animals. At this time, animals were euthanised and the tissue was processed for markers of pre-synaptic terminals, microglia, astrocytes, PrP^Sc^ and apoptotic cells in key areas known to show pathology in one or more of the three strains examined in this study.

## Methods

### Animals and stereotaxic surgery

Female C57BL/6 mice (Harlan, UK), n = 40, were group housed under standard light and temperature regimes. Food and water were available *ad libitum*, except during the glucose test when a glucose solution was substituted for water. There were 10 mice in each scrapie strain and control group, all of which were used for behavioural testing and a minimum of three from each group were used to assess pathology. All procedures were carried out under a UK Home Office license and in accordance with the Animals (Scientific Procedures) Act, 1986. Measures were taken to ameliorate the suffering of animals in all experiments. The mice (14-20g) were anaesthetised intraperitoneally with Avertin (2, 2, 2-tribromoethanol) and positioned in a stereotaxic frame (David Kopf Instruments, USA). The scalp was incised and the skull exposed. Two small holes were drilled in the skull either side of the midline to allow the bilateral injection of 1 µl of brain homogenate (10% w/v in sterile PBS). Control animals were injected with homogenate prepared from normal C57BL/6 mice (normal brain homogenate, NBH-animals). Prion animals were injected with homogenate prepared from C57BL/6 mice in the terminal stages of scrapie of the ME7, 79A or 22L strain. The ME7 strain tissue was generated in-house and 79A and 22L strain tissue was obtained from the TSE Resource Centre, Institute of Animal Health, UK. The injections were made directly into the hippocampus with coordinates measured from Bregma: anteroposterior -2.0 mm; lateral -1.7 mm; depth -1.6 mm. Injections were made with a 10 µl Hamilton syringe adapted with a 26-gauge needle.

### Behavioural testing

Mice were habituated to the burrowing and glucose tasks and from week 10 post-injection were tested weekly. Plastic cylinders, 20 cm long, 6.8 cm in diameter and closed at one end were filled with 190 g of normal diet food pellets and placed in individual cages. The open end was elevated 3 cm above the base of the cage. Mice were placed into individual cages in the late afternoon and the food remaining in the cylinders after 2 hours was weighed and the amount of food displaced or burrowed was calculated. 5% glucose solution in pre-weighed bottles was substituted for the drinking water. The bottles and burrowing tubes were reweighed the next morning and the amount of glucose drunk or pellets burrowed was calculated.

### Tissue processing and immunohistochemistry

Once statistically significant changes had been observed with the behavioural tests (13 weeks after injection; see results), animals were terminally anaesthetised with sodium pentobarbitone and transcardially perfused with heparinised saline followed by 10% formal-saline. Brains were paraffin embedded and 10 µm coronal sections through the septum, dorsal hippocampus, thalamus, raphe and cerebellum were cut on a microtome, dewaxed in xylene and rehydrated. Immunohistochemistry was performed for microglia (IBA1, Abcam, UK), glial fibrillary acidic protein (GFAP, Dako, UK), synaptophysin (SY38, Chemicon, UK) and PrP^Sc^ (6H4, Prionics, Switzerland). TUNEL staining kits were purchased from Promega, UK. Biotinylated secondary antibodies, normal sera, mouse-on-mouse (MOM) blocking kit and avidin-biotin complex were from Vector Laboratories, UK. Immunohistochemistry was carried out by the avidin-biotin-complex method (ABC) method, with 0.015% v/v hydrogen peroxide as the substrate and visualised with diaminobenzidine (DAB). Primary antibody specific modifications are detailed below.

### Synaptophysin

Rehydrated sections were treated for 30 minutes with 0.2 M boric acid, pH 9, 65 °C and incubated with 1% H_2_O_2_/PBS for 15 minutes to eliminate non-specific peroxidase activity. Sections were washed in PBS and blocked with 10% normal horse serum. Sections were incubated overnight with anti-synaptophysin (1:2000) at room temperature, washed with PBS and incubated with biotinylated horse anti-mouse secondary antibody (1:200). The DAB reaction was carried out with the addition of ammonium nickel sulphate (0.04% w/v) to enhance the intensity. The sections were dehydrated and mounted in Depex.

The density of synaptophysin staining was quantified by pixel density analysis on digitally captured images using ImageJ image analysis software (NIH, USA) using a similar method to that which we previously published [1]. In the stratum under test, areas of uniform staining were quantified by pixel density analysis. A background reading was taken from the DG granule layer (the area of highest transmittance) as an internal standard and all other transmittances were subtracted from this. Four or five sections per animal from three animals per experimental and control groups were assayed. The data was expressed as a ratio of the transmittance in the stratum radiatum to the transmittance in the adjacent stratum lacunosum moleculare.

### GFAP

Rehydrated sections were treated with 1% H_2_O_2_ to block endogenous peroxidase activity and microwaved at full power in 10 mM citrate buffer, pH 6, for 3 minutes followed by 5 minutes cooling and a further 5 minutes microwaving. Washed sections were incubated in 0.04% pepsin in 0.1 M HCl for 10 minutes. Sections were blocked with 10% normal goat serum followed by anti-GFAP, 1:2000 for 90 minutes at room temperature. Washed sections were incubated with biotinylated goat anti-rabbit (1:100). Sections were incubated with ABC and visualised with DAB. Sections were counterstained with haematoxylin, dehydrated and mounted in Depex.

The density of astrocytes was scored as 0, +, ++ or +++ in a blinded fashion with GFAP levels in NBH-animals in that region set at 0. Scoring was carried out in the lateral septum, dorsal part (LSD); the medial septum-diagonal band of Broca (MSDB), the dorsal hippocampal formation, the dorsal thalamus; dorsal raphe; median raphe; the simple lobule of the cerebellum. The results are presented relative to NBH scores.

### IBA1

Rehydrated sections were treated as above to block endogenous peroxidase activity and for antigen retrieval with the addition of 0.1% Tween in the PBS wash solution. Sections were blocked with 10% normal horse serum and incubated overnight with anti-IBA1, 1:200 at 4 °C. Sections were incubated with biotinylated horse anti-goat (1:100). The antigen was visualised and the sections mounted as above.

The density of microglia were scored as 0, +, ++ or +++ in a blinded fashion with 0 equal to the widespread relatively uniform distribution of IBA-1-positive microglia seen in sections from NBH-animals. The scoring was carried out in the same structures as above and the results are presented relative to NBH scores.

### PrP^Sc^

Rehydrated sections were autoclaved at 121 °C for 15 minutes, washed in PBS and treated with 95% formic acid to destroy PrP^C^. To avoid potential non-specific binding that can occur using a mouse monoclonal antibody, the sections were incubated with MOM blocking reagent, MOM diluent and the anti-PrP (6H4, 1:4000) was made up in diluent. Sections were incubated overnight at 4 °C. Washed sections were incubated with biotinylated anti-mouse IgG, (1:250) and the antigen was visualised and section mounted as above.

### TUNEL

Rehydrated sections were pre-incubated in equilibrium buffer for 10 minutes at 37 °C and then with incubation medium for 2 hours at 37 °C, labelling fragmented DNA with fluorescein. The reaction was stopped with sodium citrate solution, blocked with 10% normal goat serum and subsequently incubated with biotinylated goat anti-fluorescein (1:200). Sections were then processed as above. Late stage ME7 hippocampal sections were processed alongside the early stage tissue as a positive control. TUNEL positive nuclei were counted in full coronal sections of the septum (distance from Bregma: 1.10 mm to 0.62 mm); dentate gyrus, hippocampus and thalamus (-1.46 mm to -1.82 mm); dorsal and median raphe (-4.36 mm to -4.48 mm); cerebellum (-5.40 mm to -5.68 mm).

### Statistics

Behavioural data were compared by repeated measures two-way ANOVA to examine the effects of treatment group (ME7-, 79A-, 22L-, NBH-animals) and of time (10-13 weeks). Since the experiments were designed to terminate as soon as significant differences became apparent, all groups were also compared with NBH-animals with Bonferroni post-hoc tests. Ratio measurements of synaptophysin density were analysed by ANOVA with Bonferroni post-hoc tests. TUNEL counts were compared between groups by one-way ANOVA.

## Results

### Behavioural testing

**Figure 1:**
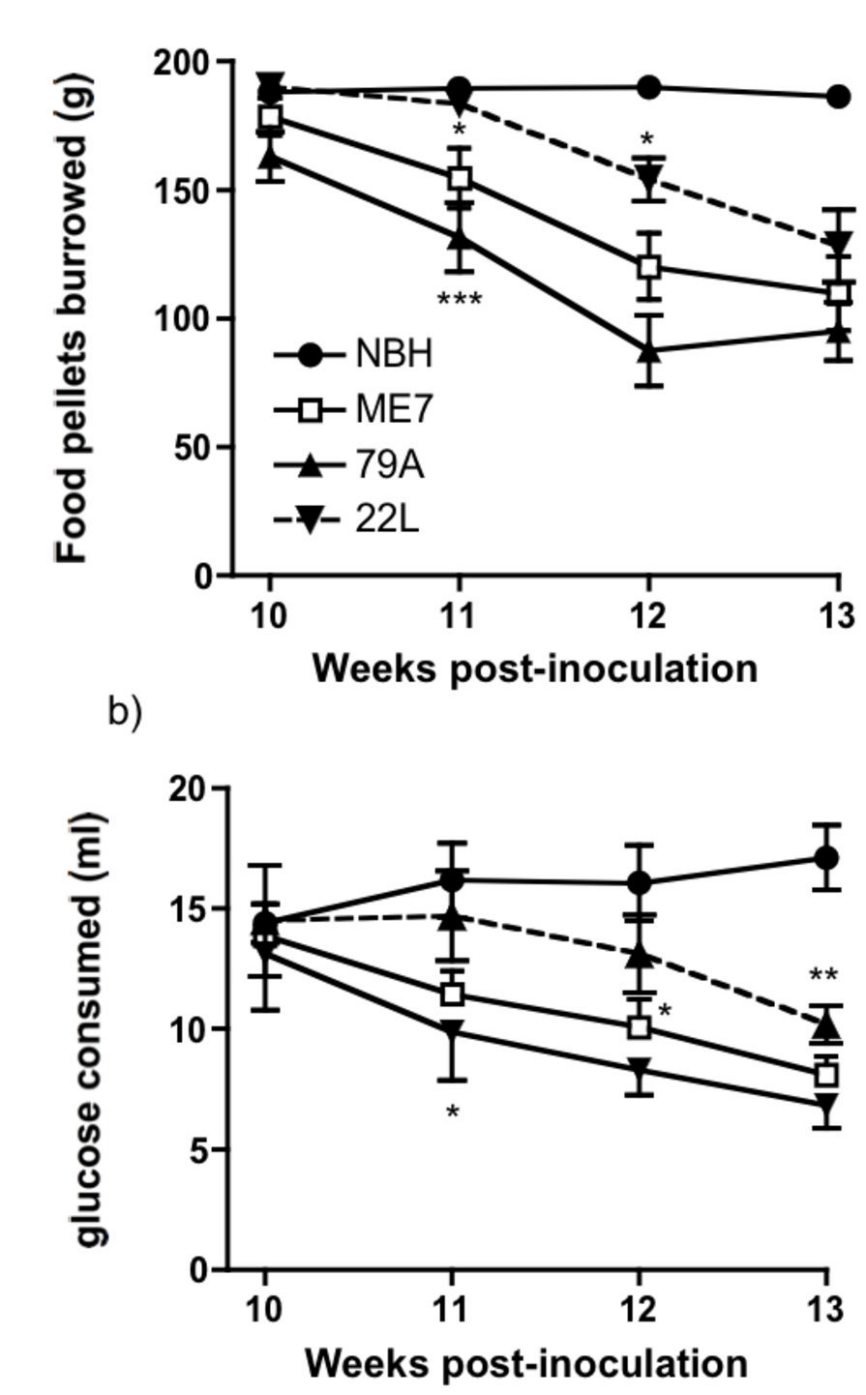
Behavioural analysis of the prion-diseased and control animals. All three strains of prion disease agent caused behavioural deficits with a similar temporal sequence. a) In a food pellet-burrowing task, the amount (weight, g) burrowed after two hours was plotted against weeks post-inoculation. b) In the glucose consumption test, the amount of 5% glucose solution drunk overnight, when singly housed, was measured. Polydipsia was established but consumption subsequently began to decline significantly in all strains with respect to NBH (n = 10 animals per strain, *P < 0.05, **P < 0.01, ***P < 0.001, Bonferroni *post hoc* test after significant effects in repeated measures two-way ANOVA).

The aim of this study was to compare neuropathological changes in the three strains once statistically significant changes on species typical behaviours had been observed in all three. The results of the burrowing and glucose tests were comparable to that seen in our previous studies [1,6]. When NBH-animals and the animals inoculated with each of 3 prion strains were compared on burrowing (Figure 1a) and glucose consumption (Figure 1b), similar patterns were observed for all three strains, with slightly differing temporal characteristics. We performed repeated measures two-way ANOVA on burrowing data, with strain (NBH, ME7, 79A and 22L) as the between subjects factor and time as the within subjects factor. This revealed main effects of time (F=49.93, df 3,108, p<0.0001) and of strain (F=15.25, df 3,36, p<0.0001) and an interaction of time and strain (F=6.68, df 9,108, p<0.0001). Bonferroni post-hoc tests revealed that ME7 and 79A were significantly different from NBH from 11 weeks (p<0.05 and <0.001 respectively) while 22L became significantly different from NBH from 12 weeks (p<0.05). By week 13 after injection, all three strains showed a highly significant decrease in the amount of pellets burrowed when compared to controls (NBH vs. ME7, 79A or 22L *P* < 0.001).

Between weeks 7 and 10 after injection, animals in all groups habituated to the glucose solution, became polydipsic and drank between 10–15 ml of a 5% glucose solution overnight. Thereafter, from week 10-13 (Figure 1b), glucose consumption decreased in all strains with respect to NBH, showing main effects of time (F=10.03. df 3,108, p<0.0001) and of strain (F=5, df 3,36, p<0.01) and an interaction of these factors (F=3.95, df 9,108, p<0.0005). Bonferroni post-hoc tests showed that differences between NBH and 22L reached significance at 11 weeks (p<0.05), ME7 was significantly different from NBH from 12 weeks (p<0.05) and 79A was significantly different to NBH from 13 weeks (p<0.01). NBH animals continued to drink large amounts of glucose solution (>15 ml).

### Synaptophysin

**Figure 2:**
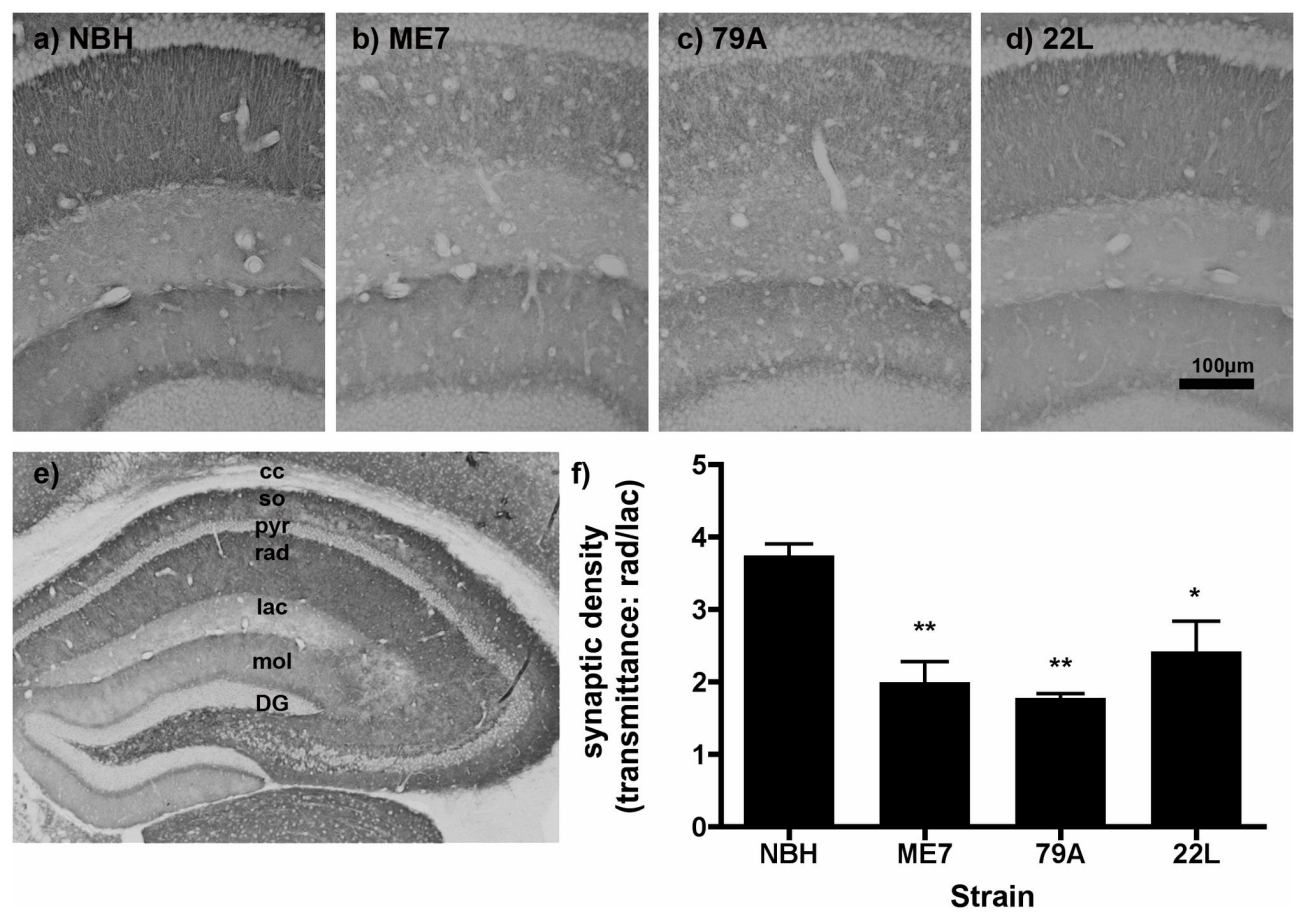
Hippocampal synaptophysin in prion-diseased and normal brain homogenate-inoculated animals. Sy38 labelling of the presynaptic marker synaptophysin in a) NBH, b) ME7, c) 79A, and d) 22L at 13 weeks post-inoculation. e) The laminar structure of the hippocampus of an NBH animal: there is particularly dense synaptophysin labelling in the stratum oriens and *radiatum* in CA1 and CA3 and at the border between the granule cells and the molecular layer of the dentate gyrus. f) There is a significant decrease in the ratio of synaptic density in the stratum radiatum versus the synaptic density in the stratum lacunosum for all three prion strains when compared to NBH animals. ** *P* < 0.01, by Bonferroni *post hoc* test after a significant one-way ANOVA (n=3-4 sections from each of 3 animals per treatment group). Abbreviations: DG, dentate gyrus; mol, molecular layer, dentate gyrus; lac, lacunosum moleculare layer, hippocampus; so, stratum oriens; rad, stratum radiatum; pyr, pyramidal cell layer, Scale bar = 100 µm.

Synaptophysin staining identifies presynaptic terminals and has been used previously to demonstrate synaptic loss in murine prion disease [1–3,5,6]. Micrographs of the laminar structure are shown for NBH (Figure 2a), ME7 (2b), 79A (2c) and 22L (2d). The full laminar structure of the hippocampus in an NBH-animal can be seen at lower power in Figure 2e, demonstrating the high density of presynaptic terminals in the stratum radiatum and stratum oriens of CA3 and CA1. This corresponds to the mossy fibre projection from the granule cells in the dentate gyrus to the pyramidal neurons in CA3 and the Schaffer collaterals from CA3 to CA1 respectively. At 13 weeks post-injection, there is disruption of the laminar structure in the CA1 region of the hippocampus and a reduction in the density of the synaptophysin staining in the stratum radiatum of all experimental groups when compared to NBH animals (Figure 2a vs. 2b-d). This appeared to be most severe in 79A and most variable in 22L but was present in all prion diseased animals. Pixel density analysis of synaptophysin labelling revealed that the ratio of transmittance in the stratum radiatum relative to the stratum lacunosum-moleculare is significantly decreased in ME7 (p<0.01), 79A (p<0.01) and in 22L (p<0.05) when compared to NBH-animals (Figure 2f; Bonferroni post-hoc comparisons to NBH after a significant one way ANOVA).

### Astrocytes

**Figure 3:**
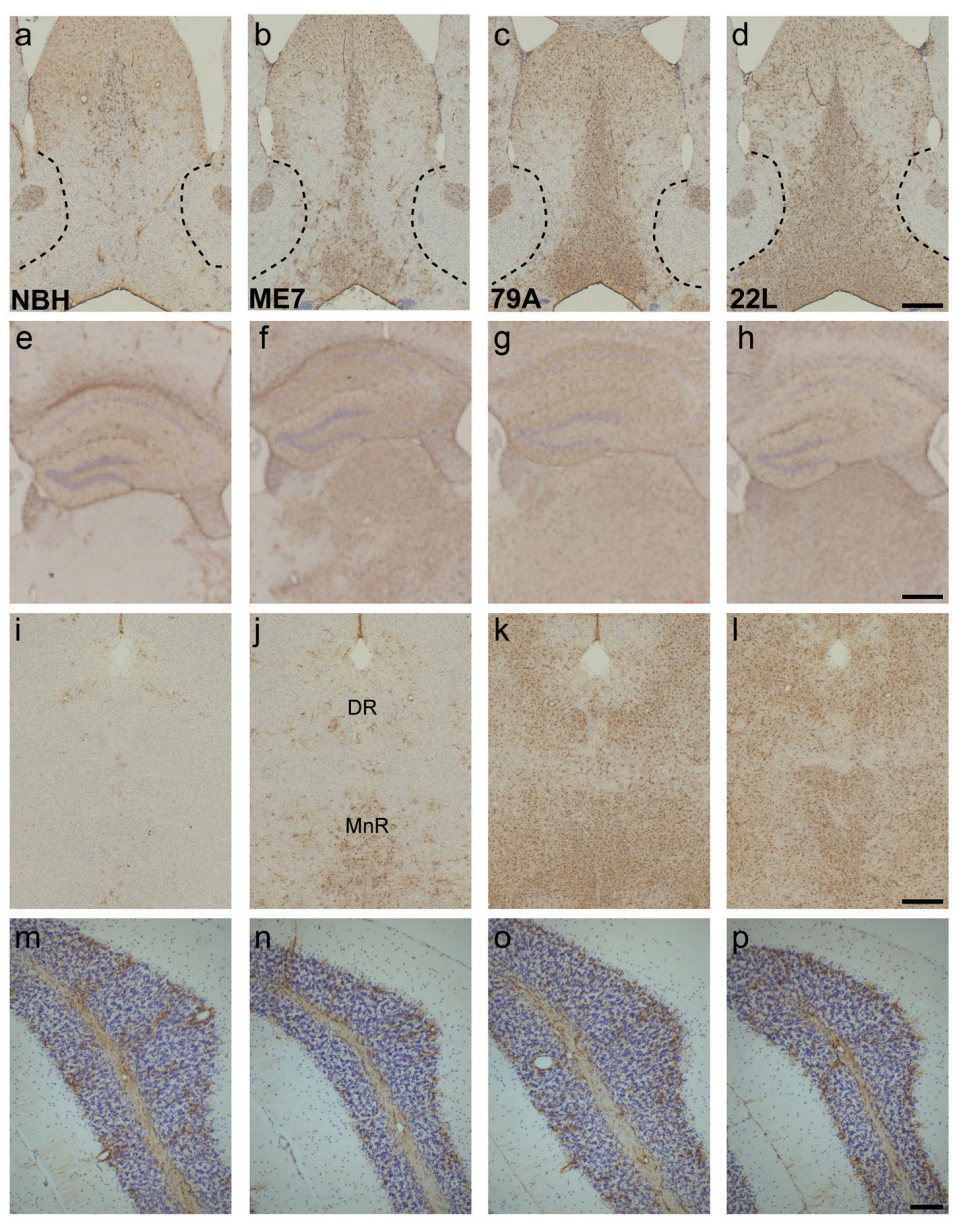
GFAP-positive astrocytes increase in prion-diseased and normal brain homogenate-inoculated animals. a–d) The number of astrocytes detectable with GFAP dramatically increases in the MSDB and the dorsal part of the lateral septum of prion animals when compared to controls (3a vs. 3b-d). e–h) GFAP-positive astrocytes were present in the NBH dorsal hippocampus (3e), and were increased in all strains with robust upregulation in ME7 and 79A animals (3f & 3g) and a more variable increase in 22L animals (3h). i–l) Only blood vessel-associated astrocytes were detected in the dorsal (DR) and median raphe (MnR) in NBH animals (3i) but GFAP was readily detectable in ME7, 79A and 22L animals (3j-l). m–p) In the simple lobule of the cerebellum the scattered and variable levels of GFAP positive astrocytes were similar in all four treatment groups. Scale bars = 500 µm a-l, 100 µm m-p.

In general, there was an increase in the density of astrocytes in selected brain regions in all three groups of prion-infected animals and the level of increase did show some strain specificity. GFAP positive astrocytes are present in the septal region of NBH-animals, however the number of astrocytes with detectable levels of GFAP has massively increased in the MSDB of prion infected animals (Figure 3a vs. 3b-d). Particularly in the 79A- and 22L-animals, this increase clearly highlights the border between the MSDB and the adjacent GFAP-negative shell of the nucleus accumbens (Figure 3c & 3d). Increased numbers of GFAP-positive astrocytes were also present in the dorsal part of lateral septum, which receives a projection from the hippocampus. In NBH-animals, GFAP positive astrocytes are detectable in the hippocampus and the dentate gyrus, whereas in the dorsal thalamus and the overlying cortex, astrocytes can only be seen around blood vessels (Figure 3e). In ME7- and 79A-animals, the hippocampus and the DG was a site of GFAP upregulation (Figure 3f & 3g). There was GFAP-positive astrocytosis in the hippocampus of 22L animals although this was more limited and more variable that in ME7 and 79A (Figure 3h). There was a dramatic increase in detectable levels of GFAP in the dorsal thalamus in all three strains compared to NBH-animals (Figure 3e vs. 3f-h), however it can be seen that in the ME7-animals there are some thalamic midline nuclei that do not have GFAP upregulation. In the dorsal and median raphe of NBH-animals, the only astrocytes with detectable levels of GFAP are around blood vessels (Figure 3i), while the dorsal and median raphe in all three prion strains showed an increase in GFAP positive astrocytes, with the greatest increase in the 22L-animals (Figure 3j–l). At 13 weeks, the level of GFAP positive astrocytes in the simple lobe of the cerebellum is similar in all four groups (Figure 3m–p). In the 22L-animals there is no or only weak up-regulation of GFAP in the vicinity of the Purkinje neurons, a cell population that is severely affected at late stage in this strain (Figure 3p). The density of GFAP positive astrocytes in the structures of interest was scored as 0, +, ++ or +++ and the results are presented relative to the scores from NBH-animals (designated 0) (Table 1).

**Table 1:** Grading of pathological features.

	**Astrocytes**				**Microglia**				**PrP^Sc^**		
Structure	**ME7**	**79A**	**22L**		**ME7**	**79A**	**22L**		**ME7**	**79A**	**22L**
Medial septum/diagonal band	+	++	++/+++		+	+	++		+	++	+++
Dentate gyrus/CA3	+	+/++	0/+		+	+/++	+		+++	++	++/+++
Hippocampus/ CA1	+/++	+/++	0/+		++	++	+		++	++	+
Thalamus	++	++	++/+++		+/++	++/+++	++/+++		++	++	++
Dorsal raphe	0/+	+	++		0	0/+	+/++		+/++	+/++	+++
Median raphe	+/++	++	++		+	+	++		++	++	+++
Cerebellum, simple lobule	0	0/+	0/+		0	0	0/+		0	0	0/+

Sections were scored as 0, +, ++, or +++ where the pattern/number of astrocytes, microglia or PrP^Sc^ in NBH is set to zero and other animalsare presented relative to NBH scores.

### Microglia

**Figure 4:**
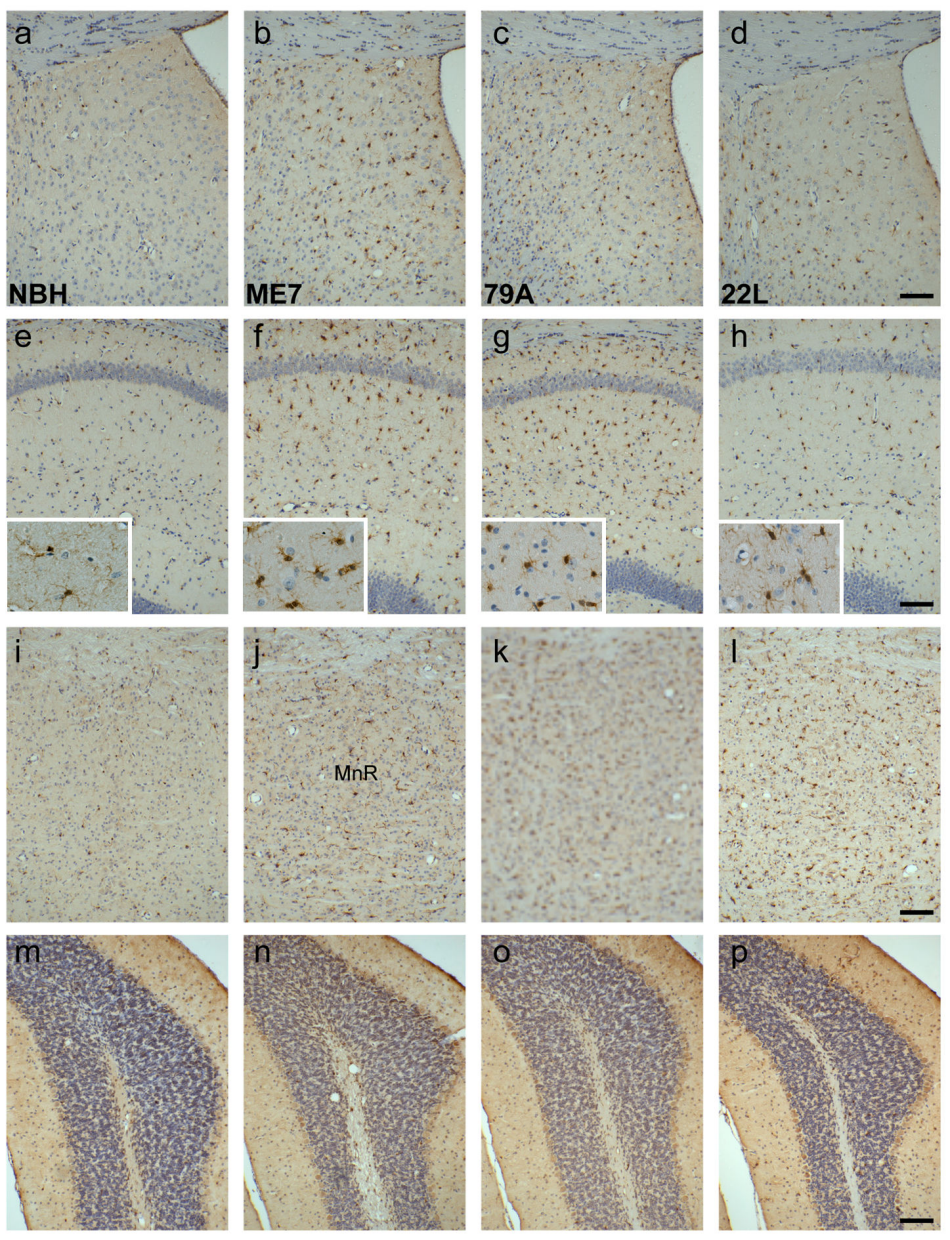
IBA1 positive microglia increase and have an activated morphology in prion animals. a–d) Compared to the sparse, uniform distribution of microglia in NBH animals, the lateral septum, dorsal part, shows an increased expression in the prion strains (4a vs. 4b-d). e–h) CA1 of the hippocampus shows a large increase of microglia with activated morphology (insets) in the ME7 and 79A strains, with a more modest increase in the 22L strain (4e vs. 4f-h). i-l): The median raphe has increased microglia in all strains (4i vs. 4j-l). m–p) microglia levels were similar in all treatment groups. Scale bars = 100 µm.

IBA-1 is constitutively expressed in microglia. In NBH-animals, immunohistochemistry for IBA1 showed microglia sparsely distributed throughout the brain. We observed a large increase in the numbers of microglia in a number of regions in the prion-diseased animals. The lateral septum, which receives a direct projection from the hippocampus showed significant increases in IBA-1-positive microglia in the ME7- and 79A-animals (Figure 4b & 4c) and more limited activation in 22L (Figure 4d). This is consistent with the more robust microglial activation in the hippocampus of ME7 and 79A (Figure 4f & 4g) with respect to the more limited labelling in 22L (Figure 4h). Microglia in prion strains show a more condensed morphology with respect to the ramified morphology in NBH animals (insets). In the median raphe, there was an increase in the number of microglia in all prion animals (Figure 4j–l) although this increase was most prominent in the 79A- and 22L-animals (Figure 4k & 4l). In the cerebellum, typified by the simple lobule, there was no increase in the density of microglia in the ME7- and 79A-animals compared to NBH-animals (Figure 4m vs. 4n & 4o). There was very limited and variable increase in the number of microglia in 22L-animals (Figure 4p). The density of microglia was scored as 0, +, ++ or +++ and the results were presented relative to the scores from NBH-animals (designated 0) (Table 1).

### PrP^Sc^

**Figure 5:**
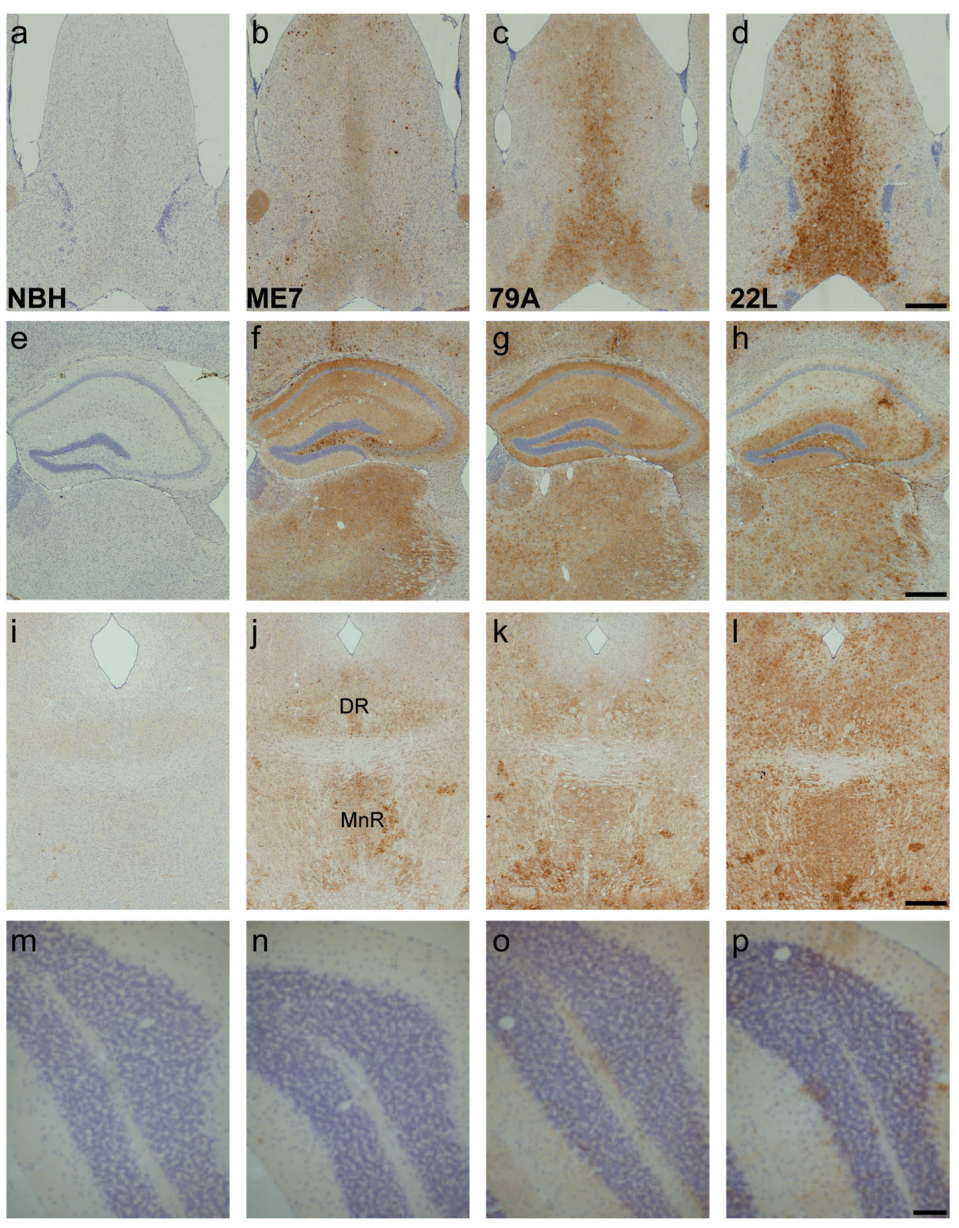
PrP^Sc^ deposition is widespread at 13 weeks in the prion-infected animals, but there are strain specific differences. a–d) PrP^Sc^ is deposited in a granular form or in large, dense plaques. There is no PrP^Sc^ deposition in the control animals. e–h) The dentate gyrus and the hippocampus have a high PrP^Sc^ load in all three strains and none in the NBH animals. There is a strain difference in the CA1 field with deposition in the 22L strain predominantly confined to the stratum oriens and *radiatum*. i–l) PrP^Sc^ is deposited in the median and dorsal raphe, especially in the 22L strain. m–p) In the simple lobule of ME7 and 79A animals PrP^Sc^ deposition is indistinguishable from that seen in NBH-animals. There is a small amount of PrP^Sc^ around the Purkinje cells of the 22L mice. Scale bars = 500 µm a-l, 100 µm m-p.

In all prion disease animals, at 13 weeks after injection, there were deposits of PrP^Sc^ protein present in the regions of the brain examined (Figures 5b–d, 5f–h, 5j–l & p) and no deposition in the control animals (Figures 5a, 5e, 5i & 5m). In the septum of 79A-animals, there are predominantly granular deposits in the MSDB (Figure 5c) whereas in 22L-animals there is a large deposition of PrP^Sc^ in the MSDB and the lateral septum (Figure 5d). In the hippocampus, there is prominent deposition in the polymorphic layer of the dentate gyrus and the stratum lucidum of CA3 in the hippocampus for all three strains and granular deposition in the other layers of the dentate gyrus (Figure 5f–h). In contrast to the ME7- and 79A-animals, the CA1 region of the 22L-animals has a sparse deposition of PrP^Sc^ in the stratum radiatum and stratum oriens (Figure 5h) compared to its marked deposition in the dentate gyrus of these animals. PrP^Sc^ deposition in the dorsal and median raphe is found in all three strains (Figure 5j–l) and the deposition is very extensive in the 22L-animals (Figure 5l). In the simple lobe of the cerebellum, the ME7- and 79A-animals are indistinguishable from control animals (Figure 5m vs. 5n & 5o). In 22L-animals there is a small and variable amount of PrP^Sc^ deposition around the Purkinje neurons (Figure 5p). A summary of these assessments is shown in table 1.

### Cell death

TUNEL labelling was performed in order to identify evidence of early cell death. In the areas evaluated in this study cell death was negligible. Apoptotic cells were very rare (≤1 per 10 µm coronal section) but were readily detectable in the positive control tissue (hippocampus from 18 week ME7-animals). There was, however, a modest increase in the number of apoptotic cells in the granule layer cerebellum of 79A- and 22L-animals (4 and 12 cells per coronal section respectively).

## Discussion

In this study we have investigated the pathological substrates that associate with the early behavioural deficits seen following intracerebral inoculation with three different strains of prion agent. In our previous studies ME7-, 79A- and 22L-animals showed a decline in burrowing performance and glucose consumption when compared to NBH-animals many weeks prior to the onset of overt clinical symptoms [6] but showed divergent late stage pathology. Here we showed, at early stages, just when behavioural impairments first become apparent, the presynaptic marker synaptophysin demonstrates a loss of presynaptic terminals in the CA1 stratum radiatum of all three strains. By contrast, TUNEL-positive apoptotic cells were essentially absent at this time point. Synaptic loss in the dorsal hippocampus precedes neuronal death and is temporally associated with early behavioural deficits in three neuropathologically distinct prion strains when intrahippocampally inoculated.

### Technical considerations

One might argue that the injection of 1µl of 10% w/v infected brain homogenate into the dorsal hippocampus might be predicted to induce hippocampal pathology and dysfunction. However, it is clear that this dysfunction does not occur until 10-13 weeks post inoculation and is not seen in NBH-animals, so cannot be a result of surgery-induced hippocampal injury. Moreover, prion infectivity is well distributed through the brain parenchyma in this time. Many protocols still administer prion-infected homogenate no more precisely than ‘intracerebrally’ or ‘in the right cerebral hemisphere’ and with large volumes (≥20 µl) [14,15]. Such volumes rapidly spread considerable distances along the brain vasculature and white matter tracts [16] and indeed into the circulation [17]. These common protocols exert little control over where the inoculum goes and more precise protocols offer a greater degree of standardisation. The intra-hippocampal injection procedure leads to minimal direct damage to the hippocampus [18] and animals displaying ‘clinical signs’ using this surgical protocol developed pathology [6] faithful to the published profiles documented after homogenate injection into the frontal cortex [4,5]. Clearly intraperitoneal inoculation induces substantially different survival times and pathological profiles compared to intracerebral inoculation [15] and intracerebral injections in the cerebellum, close to presumed brainstem ‘clinical target areas’ [19,20], can also produce significantly different patterns of PrP^Sc^ deposition and vacuolation [21] indicating that site of disease initiation can influence disease course. However, our protocol does induce disease progression, survival times and late stage pathology consistent with other, less precise, forebrain-initiated surgical protocols. Furthermore, robust behavioural deficits in the burrowing task and the glucose consumption test are seen in animals with striatal or cerebellar injection at these early time points with no overt clinical symptoms [16].

The results show significant areas of similarity between these strains as well as features that distinguish them from each other. As previous terminal stages studies would have predicted, there is some cerebellar tropism in the 22L strain that is not obvious in ME7 and 79A strains, and indeed 22L shows somewhat weaker synaptic loss, microglial activation and PrP^Sc^ deposition in the CA1 of the hippocampus with respect to the dentate gyrus/CA3 region. However, that 22L can produce CA1 synaptic loss is significant, and this has now been confirmed by electron microscopy studies [22]. On the whole 79A produces rather similar early pathology to ME7. As we reported in terminal stages of these 3 strains [6], it is once again evident from microglial, astrocytic and PrP^Sc^ immunolabelling that the thalamus is significantly affected in all 3 strains and the emergence of this pathology, which cuts across several different ventrobasal nuclei of the thalamus, requires further study. Combining the data arising from this study with prior studies with different strains and different sites of inoculation, it appears that these multiple strains can produce limbic system and thalamic pathology given appropriate exposure to the inoculum. Though key clinical target areas have not been definitively identified, it appears that inoculation close to the brainstem will lead to death of the animal before pathology can become evident in some of the regions identified in this forebrain-initiated study.

### Synaptic loss preceding neurodegeneration

The most consistent finding of this study was that the behavioural deficits were associated with a decrease in synaptophysin immunostaining in the CA1 field of the hippocampus in all three prion strains. Of particular note is the synaptic loss in the 22L strain, which does not have CA1 pyramidal cell death even at the later stages of the disease [6]. This advances the previous finding that behavioural changes and hippocampal synaptic loss in ME7 animals occurs several weeks prior to neuronal cell death [1]. Jeffrey and colleagues noted the first axon terminal degeneration and synaptic loss at 34-42% of the incubation period (IP) in ME7 in C57BL/6J **x** VM/Dk mice, but no cell death in CA1 until 72% IP [2]. In the current study, the first behavioural deficits were found at 11 weeks and both burrowing deficits and decreased glucose consumption were present in all three strains by 13 weeks. At this time all animals were euthanized. Synaptic loss was present in all three strains and hippocampal or limbic system neuronal loss was absent in all three strains. Although a decrease in synaptic proteins such as synapsin, SNAP-25 and synaptophysin has been demonstrated in post mortem CJD cerebral cortex and cerebellum [10,23,24] and in the RML mouse model [25], these analyses were undertaken at a time when there was extensive neuronal death and thus severe synaptic loss would be expected. The current study demonstrates clearly that, if exposed to the prion infectivity, all three strains ME7, 79A and 22L show robust synaptic loss in the hippocampus and early behavioural deficits. It has been shown that hippocampal lesions have a profound affect on the burrowing behavioural assay [26] and while we show a clear correlation between hippocampal synaptic loss and the onset of a burrowing deficit in these prion strains we cannot exclude the possible involvement of other brain structures. The thalamus, in particular, is clearly affected in all three strains [6]. Glucose consumption could be predicted to involve other brain structures such as the lateral hypothalamus [27], and some authors have reported vacuolation in this region in ME7 and 79A strains [28]. Nothwithstanding possible involvement of multiple brain structures, in neurofilament heavy chain-Cre mice inoculated with the RML-strain of prion disease, it has been demonstrated that synaptic loss can be reversed by switching off neuronal expression of PrP^c^ and this also reverses early behavioural/cognitive dysfunction [3]. Furthermore, prevention of neuronal apoptosis by induction of prion disease in Bax-/- mice had no impact on time to onset of clinical signs or time of death and had no impact on synaptic loss [29]. Collectively these data suggest that synaptic loss is indeed an early component of prion pathology that is correlated with behavioural/clinical changes and our current data indicate that the described synaptic changes are likely to occur in many different prion strains after intracerebral inoculation.

Synaptic degeneration has also been identified in other CNS neurodegenerative disorders. Synaptic loss is regarded as the best correlate of cognitive decline [30] in Alzheimer’s disease and, congruent with this, synaptic loss is present but less prominent in mild cognitive impairment (MCI) and in transgenic AD models [31–33]. Synaptic loss has also been documented as an early event, preceding most neuronal death in Huntington’s disease and in motor neuron disease, correlating with the deterioration of mental and motor functions [34–36]. Thus synaptic loss is a key feature of many chronic neurodegenerative diseases and occurs before neuronal death in many disease states. Little is known about the mechanisms by which presynaptic terminals degenerate in any of these conditions and the robust and reproducible synaptic loss in the stratum radiatum in these prion strains presents a tractable model to study aspects of the cellular and molecular mechanisms that underpin this process.

### Mechanisms of synaptic loss

Biochemical studies of the hippocampus of ME7-animals showed that proteins of the presynaptic terminal compartment were the first to decline and in particular those of the synaptic vesicles [37]. There are also reports of significant cortical dendritic spine alterations in the RML strain [38]. However, recent electron microscopy studies with ME7 animals have revealed that the loss of the integrity of the synaptic vesicles is the first apparent morphological feature of synapse degeneration in the stratum radiatum (Siskova et al., 2009). The electron dense pre-synaptic element remains in close apposition to the postsynaptic density of dendritic spine which becomes progressively wrapped around degenerating presynaptic elements [13]. Identical profiles are seen in the stratum radiatum of 22L-animals at 12 weeks after disease initiation [22]. Simultaneously, synaptic swelling/hypertrophy occurs in apparently intact presynaptic terminals [39], which may be a precursor to, or a mechanism to compensate for, synaptic loss.

PrP^C^ is highly concentrated in presynaptic terminals where it co-localises with synaptophysin and is highly prevalent in brain structures associated with synaptic plasticity [40–43] and PrP^C^ knockout mice show deficiencies in GABA_A_ receptor mediated fast inhibition and impaired long-term potentiation (LTP) [44]. Those data suggest that the loss of normal PrP^C^ function may contribute to synaptic dysfunction and ultimately to neuronal loss. However, it has been shown that blocking synaptic activity in the ME7 model, using botulinum neurotoxin A, does not induce any additional or more rapid synaptic loss in these animals [45]. Furthermore there are studies to show that PrP^Sc^ generated by astrocytes in the absence of neuronal expression of PrP^C^ still leads to neuronal degeneration [46]. There remains no clear understanding of the physiological function of PrP^C^, but the accumulation of oligomeric aggregates of the aberrant PrP^Sc^ [47] initiates neuronal degeneration at the pre-synaptic terminal in a number of different prion strains by mechanisms that have yet to be unravelled.

## Conclusion

Synaptopathy in the hippocampal formation is common to three strains of prion disease upon intra-hippocampal inoculation and is an early pathological feature of the disease. Since these features occur in association with behavioural changes but significantly before neuronal death, this indicates that synaptic loss is a key neuropathological feature of multiple strains of prion disease. Thus, these prion disease models offer tractable routes into elucidation of molecular and cellular events underpinning synaptic loss. Further studies of these models may provide insights into synaptic degeneration that may be exploited in multiple disease states.

## References

[1] Cunningham C, Deacon R, Wells H, Boche D, Waters S et al. (2003) Synaptic changes characterize early behavioural changes in the ME7 model of murine prion disease. Eur J Neurosci 17: 2147-2155. doi:10.1046/j.1460-9568.2003.02662.x. PubMed: 12786981. 12786981 10.1046/j.1460-9568.2003.02662.x

[2] Jeffrey M, Halliday WG, Bell J, Johnston AR, MacLeod NK et al. (2000) Synapse loss associated with abnormal PrP precedes neuronal degeneration in the scrapie-infected murine hippocampus. Neuropathol Appl Neurobiol 26: 41-54. doi:10.1046/j.1365-2990.2000.00216.x. PubMed: 10736066. 10736066 10.1046/j.1365-2990.2000.00216.x

[3] Mallucci GR, White MD, Farmer M, Dickinson A, Khatun H et al. (2007) Targeting cellular prion protein reverses early cognitive deficits and neurophysiological dysfunction in prion-infected mice. Neuron 53: 325-335. doi:10.1016/j.neuron.2007.01.005. PubMed: 17270731. 17270731 10.1016/j.neuron.2007.01.005

[4] Bruce ME, McConnell I, Fraser H, Dickinson AG (1991) The disease characteristics of different strains of scrapie in Sinc congenic mouse lines: implications for the nature of the agent and host control of pathogenesis. J Gen Virol 72: 595-603. doi:10.1099/0022-1317-72-3-595. PubMed: 1672371. 1672371 10.1099/0022-1317-72-3-595

[5] Fraser H, Dickinson AG (1968) The sequential development of the brain lesion of scrapie in three strains of mice. J Comp Pathol 78: 301-311. doi:10.1016/0021-9975(68)90006-6. PubMed: 4970192. 4970192 10.1016/0021-9975(68)90006-6

[6] Cunningham C, Deacon RM, Chan K, Boche D, Rawlins JN et al. (2005) Neuropathologically distinct prion strains give rise to similar temporal profiles of behavioral deficits. Neurobiol Dis 18: 258-269. doi:10.1016/j.nbd.2004.08.015. PubMed: 15686954. 15686954 10.1016/j.nbd.2004.08.015

[7] Betmouni S, Deacon RMJ, Rawlins JNP, Perry VH (1999) Behavioural consequences of prion disease targeted to the hippocampus in a mouse model of scrapie. Psychobiology 27(1): 63-71.

[8] Deacon RM, Raley JM, Perry VH, Rawlins JN (2001) Burrowing into prion disease. Neuroreport 12: 2053-2057. doi:10.1097/00001756-200107030-00052. PubMed: 11435945. 11435945 10.1097/00001756-200107030-00052

[9] Guenther K, Deacon RM, Perry VH, Rawlins JN (2001) Early behavioural changes in scrapie-affected mice and the influence of dapsone. Eur J Neurosci 14: 401-409. doi:10.1046/j.0953-816x.2001.01645.x. PubMed: 11553290. 11553290 10.1046/j.0953-816x.2001.01645.x

[10] Clinton J, Forsyth C, Royston MC, Roberts GW (1993) Synaptic degeneration is the primary neuropathological feature in prion disease: a preliminary study. Neuroreport 4: 65-68. doi:10.1097/00001756-199301000-00017. PubMed: 8453038. 8453038 10.1097/00001756-199301000-00017

[11] Kitamoto T, Shin RW, Doh-ura K, Tomokane N, Miyazono M et al. (1992) Abnormal isoform of prion proteins accumulates in the synaptic structures of the central nervous system in patients with Creutzfeldt-Jakob disease. Am J Pathol 140: 1285-1294. PubMed: 1351366. 1351366 PMC1886543

[12] Sikorska B, Liberski PP, Giraud P, Kopp N, Brown P (2004) Autophagy is a part of ultrastructural synaptic pathology in Creutzfeldt-Jakob disease: a brain biopsy study. Int J Biochem Cell Biol 36: 2563-2573. doi:10.1016/j.biocel.2004.04.014. PubMed: 15325593. 15325593 10.1016/j.biocel.2004.04.014

[13] Sisková Z, Page A, O’Connor V, Perry VH (2009) Degenerating synaptic boutons in prion disease: microglia activation without synaptic stripping. Am J Pathol 175: 1610-1621. doi:10.2353/ajpath.2009.090372. PubMed: 19779137. 19779137 10.2353/ajpath.2009.090372 PMC2751557

[14] Bremer J, Heikenwalder M, Haybaeck J, Tiberi C, Krautler NJ et al. (2009) Repetitive immunization enhances the susceptibility of mice to peripherally administered prions. PLOS ONE 4: e7160. doi:10.1371/journal.pone.0007160. PubMed: 19779609. 19779609 10.1371/journal.pone.0007160 PMC2744926

[15] Langevin C, Andréoletti O, Le Dur A, Laude H, Béringue V (2011) Marked influence of the route of infection on prion strain apparent phenotype in a scrapie transgenic mouse model. Neurobiol Dis 41: 219-225. doi:10.1016/j.nbd.2010.09.010. PubMed: 20875860. 20875860 10.1016/j.nbd.2010.09.010

[16] Combrinck MI, Betmouni S, Perry VH, Cunningham C (2001) Injection site does not alter progression in the ME7 model of murine scrapie. J Neuroimmunol 118 (supplement): 107-116.

[17] Kimberlin RH, Walker CA (1979) Pathogenesis of mouse scrapie: dynamics of agent replication in spleen, spinal cord and brain after infection by different routes. J Comp Pathol 89: 551-562. doi:10.1016/0021-9975(79)90046-X. PubMed: 120379. 120379 10.1016/0021-9975(79)90046-x

[18] Betmouni S, Perry VH (1999) The acute inflammatory response in CNS following injection of prion brain homogenate or normal brain homogenate. Neuropathol Appl Neurobiol 25: 20-28. PubMed: 10194772. 10194772 10.1046/j.1365-2990.1999.00153.x

[19] Kimberlin RH, Walker CA (1986) Pathogenesis of scrapie (strain 263K) in hamsters infected intracerebrally, intraperitoneally or intraocularly. J Gen Virol 67(2): 255-263. doi:10.1099/0022-1317-67-2-255. 3080549 10.1099/0022-1317-67-2-255

[20] Kimberlin RH, Walker CA (1988) Pathogenesis of experimental scrapie. Ciba Found Symp 135: 37-62. PubMed: 3137002. 3137002 10.1002/9780470513613.ch4

[21] Kim YS, Carp RI, Callahan S, Wisniewski HM (1990) Incubation periods and histopathological changes in mice injected stereotaxically in different brain areas with the 87V scrapie strain. Acta Neuropathol (Berl) 80: 388-392. doi:10.1007/BF00307692. PubMed: 2122631. 2122631 10.1007/BF00307692

[22] Šišková Z, Reynolds RA, O’Connor V, Perry VH (2013) Brain region specific pre-synaptic and post-synaptic degeneration are early components of neuropathology in prion disease. PLOS ONE 8: e55004. doi:10.1371/journal.pone.0055004. PubMed: 23383030. 23383030 10.1371/journal.pone.0055004 PMC3559345

[23] Ferrer I, Rivera R, Blanco R, Martí E (1999) Expression of proteins linked to exocytosis and neurotransmission in patients with Creutzfeldt-Jakob disease. Neurobiol Dis 6: 92-100. doi:10.1006/nbdi.1998.0226. PubMed: 10343324. 10343324 10.1006/nbdi.1998.0226

[24] Ferrer I, Puig B, Blanco R, Martí E (2000) Prion protein deposition and abnormal synaptic protein expression in the cerebellum in Creutzfeldt-Jakob disease. Neuroscience 97: 715-726. doi:10.1016/S0306-4522(00)00045-2. PubMed: 10842016. 10842016 10.1016/s0306-4522(00)00045-2

[25] Sisó S, Puig B, Varea R, Vidal E, Acín C et al. (2002) Abnormal synaptic protein expression and cell death in murine scrapie. Acta Neuropathol (Berl) 103: 615-626. doi:10.1007/s00401-001-0512-6. PubMed: 12012094. 12012094 10.1007/s00401-001-0512-6

[26] Deacon RM, Bannerman DM, Kirby BP, Croucher A, Rawlins JN (2002) Effects of cytotoxic hippocampal lesions in mice on a cognitive test battery. Behav Brain Res 133: 57-68. doi:10.1016/S0166-4328(01)00451-X. PubMed: 12048174. 12048174 10.1016/s0166-4328(01)00451-x

[27] Hetherington AW, Ranson SW (1942) The spontaneous activity and food intake of rats with hypothalamic lesions. Am J Physiol 136: 609-617.

[28] Outram GW, Fraser H, Wilson DT (1973) Scrapie in mice. Some effects on the brain lesion profile of ME7 agent due to genotype of donor, route of injection and genotype of recipient. J Comp Pathol 83: 19-28. doi:10.1016/0021-9975(73)90023-6. PubMed: 4199907. 4199907 10.1016/0021-9975(73)90023-6

[29] Chiesa R, Piccardo P, Dossena S, Nowoslawski L, Roth KA et al. (2005) Bax deletion prevents neuronal loss but not neurological symptoms in a transgenic model of inherited prion disease. Proc Natl Acad Sci U S A 102: 238-243. doi:10.1073/pnas.0406173102. PubMed: 15618403. 15618403 10.1073/pnas.0406173102 PMC544044

[30] Terry RD, Masliah E, Salmon DP, Butters N, DeTeresa R et al. (1991) Physical basis of cognitive alterations in Alzheimer’s disease: synapse loss is the major correlate of cognitive impairment. Ann Neurol 30: 572-580. doi:10.1002/ana.410300410. PubMed: 1789684. 1789684 10.1002/ana.410300410

[31] Coleman P, Federoff H, Kurlan R (2004) A focus on the synapse for neuroprotection in Alzheimer disease and other dementias. Neurology 63: 1155-1162. doi:10.1212/01.WNL.0000140626.48118.0A. PubMed: 15477531. 15477531 10.1212/01.wnl.0000140626.48118.0a

[32] Games D, Adams D, Alessandrini R, Barbour R, Berthelette P et al. (1995) Alzheimer-type neuropathology in transgenic mice overexpressing V717F beta-amyloid precursor protein. Nature 373: 523-527. doi:10.1038/373523a0. PubMed: 7845465. 7845465 10.1038/373523a0

[33] Mucke L, Masliah E, Yu GQ, Mallory M, Rockenstein EM et al. (2000) High-level neuronal expression of abeta 1-42 in wild-type human amyloid protein precursor transgenic mice: synaptotoxicity without plaque formation. J Neurosci 20: 4050-4058. PubMed: 10818140. 10818140 10.1523/JNEUROSCI.20-11-04050.2000 PMC6772621

[34] Li JY, Plomann M, Brundin P (2003) Huntington’s disease: a synaptopathy? Trends Mol Med 9: 414-420. doi:10.1016/j.molmed.2003.08.006. PubMed: 14557053. 14557053 10.1016/j.molmed.2003.08.006

[35] Nagao M, Misawa H, Kato S, Hirai S (1998) Loss of cholinergic synapses on the spinal motor neurons of amyotrophic lateral sclerosis. J Neuropathol Exp Neurol 57: 329-333. doi:10.1097/00005072-199804000-00004. PubMed: 9600225. 9600225 10.1097/00005072-199804000-00004

[36] Sasaki S, Maruyama S (1994) Decreased synaptophysin immunoreactivity of the anterior horns in motor neuron disease. Acta Neuropathol 87: 125-128. doi:10.1007/BF00296180. PubMed: 8171961. 8171961 10.1007/BF00296180

[37] Gray BC, Siskova Z, Perry VH, O’Connor V (2009) Selective presynaptic degeneration in the synaptopathy associated with ME7-induced hippocampal pathology. Neurobiol Dis 35: 63-74. doi:10.1016/j.nbd.2009.04.001. PubMed: 19362593. 19362593 10.1016/j.nbd.2009.04.001

[38] Fuhrmann M, Mitteregger G, Kretzschmar H, Herms J (2007) Dendritic pathology in prion disease starts at the synaptic spine. J Neurosci 27: 6224-6233. doi:10.1523/JNEUROSCI.5062-06.2007. PubMed: 17553995. 17553995 10.1523/JNEUROSCI.5062-06.2007 PMC6672160

[39] Sisková Z, Sanyal NK, Orban A, O’Connor V, Perry VH (2010) Reactive hypertrophy of synaptic varicosities within the hippocampus of prion-infected mice. Biochem Soc Trans 38: 471-475. doi:10.1042/BST0380471. PubMed: 20298205. 20298205 10.1042/BST0380471

[40] Fournier JG, Escaig-Haye F, Grigoriev V (2000) Ultrastructural localization of prion proteins: physiological and pathological implications. Microsc Res Tech 50: 76-88. doi:10.1002/1097-0029(20000701)50:1. PubMed: 10871551. 10871551 10.1002/1097-0029(20000701)50:1<76::AID-JEMT11>3.0.CO;2-#

[41] Haeberlé AM, Ribaut-Barassin C, Bombarde G, Mariani J, Hunsmann G et al. (2000) Synaptic prion protein immuno-reactivity in the rodent cerebellum. Microsc Res Tech 50: 66-75. doi:10.1002/1097-0029(20000701)50:1. PubMed: 10871550. 10871550 10.1002/1097-0029(20000701)50:1<66::AID-JEMT10>3.0.CO;2-3

[42] Herms J, Tings T, Gall S, Madlung A, Giese A et al. (1999) Evidence of presynaptic location and function of the prion protein. J Neurosci 19: 8866-8875. PubMed: 10516306. 10516306 10.1523/JNEUROSCI.19-20-08866.1999 PMC6782778

[43] Salès N, Rodolfo K, Hässig R, Faucheux B, Di Giamberardino L et al. (1998) Cellular prion protein localization in rodent and primate brain. Eur J Neurosci 10: 2464-2471. doi:10.1046/j.1460-9568.1998.00258.x. PubMed: 9749773. 9749773 10.1046/j.1460-9568.1998.00258.x

[44] Collinge J, Whittington MA, Sidle KC, Smith CJ, Palmer MS et al. (1994) Prion protein is necessary for normal synaptic function. Nature 370: 295-297. doi:10.1038/370295a0. PubMed: 8035877. 8035877 10.1038/370295a0

[45] Caleo M, Restani L, Vannini E, Siskova Z, Al-Malki H et al. (2012) The role of activity in synaptic degeneration in a protein misfolding disease, prion disease. PLOS ONE 7: e41182. doi:10.1371/journal.pone.0041182. PubMed: 22815961. 22815961 10.1371/journal.pone.0041182 PMC3397974

[46] Jeffrey M, Goodsir CM, Race RE, Chesebro B (2004) Scrapie-specific neuronal lesions are independent of neuronal PrP expression. Ann Neurol 55: 781-792. doi:10.1002/ana.20093. PubMed: 15174012. 15174012 10.1002/ana.20093

[47] Solomon IH, Schepker JA, Harris DA (2010) Prion neurotoxicity: insights from prion protein mutants. Curr Issues Mol Biol 12: 51-61. PubMed: 19767650. 19767650 PMC4821541

